# The development of BNST intrinsic functional connectivity from 8 to 23 years of age: A PNC cohort study

**DOI:** 10.1016/j.dcn.2025.101661

**Published:** 2025-12-19

**Authors:** Elizabeth A. Flook, Nicole L. Zabik, Brandee Feola, Baxter Rogers, Jennifer Urbano Blackford

**Affiliations:** a Department of Psychiatry, Hospital of the University of Pennsylvania, Philadelphia, PA, USA; b Munroe-Meyer Institute for Genetics and Rehabilitation, University of Nebraska Medical Center, Omaha, NE, USA; c Department of Psychiatry and Behavioral Sciences, Vanderbilt University Medical Center, Nashville, TN, USA; d Department of Radiology and Radiological Sciences, Institute of Imaging Science, Vanderbilt University Medical Center, Nashville, TN, USA; e Department of Biomedical Engineering, Vanderbilt University, Nashville, TN, USA

**Keywords:** Extended amygdala, BNST, Developmental connectivity, Intrinsic connectivity, Resting-state fMRI, BNST development

## Abstract

The bed nucleus of the stria terminalis (BNST) is a small subcortical region that plays a critical role in a wide array of functions, including emotion processing, reward processing, and social interactions. The BNST intrinsic functional network has been well characterized in adults. Despite evidence that BNST connectivity changes during development, maturation of the BNST network has been understudied. To address this gap, we investigated age-related changes in BNST intrinsic connectivity in youth aged 8 – 23 years using resting state functional magnetic resonance imaging scans from the Philadelphia Neurodevelopmental Cohort (PNC), a large cross-sectional dataset. We measured intrinsic connectivity within a BNST network and across the whole brain, testing for effects of age, sex, and age x sex. The BNST ROI network analysis revealed a significant decrease with age for BNST-hypothalamus connectivity and, in boys, BNST-amygdala connectivity. The whole-brain results showed that BNST connectivity was largely established by middle childhood, though there were notable increases in BNST connectivity with motor and planning regions and decreases with age in BNST-subcortical connectivity. These data suggest a shift from subcortical to control-related BNST connectivity with age during this dynamic maturational window.

## Highlights

• BNST intrinsic connectivity is largely established by middle childhood.
• With increasing age, BNST connectivity shifted from subcortical to control (motor and planning) regions.
• With increasing age, BNST-occipital cortex connectivity strengthened in boys and weakened in girls.

## 1 Introduction

The bed nucleus of the stria terminalis (BNST) is a small subcortical region that is connected to the amygdala *via* the stria terminalis and is considered part of the extended amygdala (Alheid and Heimer, 1988). The BNST has diverse connections with regions across the brain stem, subcortex, and cortex, which highlights the BNST’s potential importance as a network hub (Avery et al., 2014, Dong et al., 2000, Dong and Swanson, 2006, Krüger et al., 2015, Weller and Smith, 1982, Flook et al., 2020). Consistent with its diverse connections, the BNST has emerged as a critical brain region for a wide array of functions underlying emotion processing, reward processing, social behaviors, hormone production, learning, and feeding (for reviews see (Ch’ng et al., 2018, Crestani et al., 2013, Davis et al., 2010). Most notably, the BNST has a prominent role in modulating responses to threatening or stressful events (e.g Davis et al., 2010, Centanni et al., 2019). The BNST is also a sexually dimorphic region, as both male rats (Hines et al., 1992) and human men (Allen and Gorski, 1990) have larger BNST volumes (however see Berry et al., 2024).

Research in humans has characterized a BNST functional network in adults using intrinsic (i.e., resting state) functional connectivity. Intrinsic functional connectivity is measured using functional magnetic resonance imaging (fMRI) while an individual is ‘at rest’—that is, not engaged in a task—and reflects the brain’s intrinsic functional architecture (Biswal, 2012). Intrinsic functional architecture likely supports important communication pathways that maintain brain homeostasis, integrates information across the brain, and facilitates readiness for performing tasks. Studies in adults show BNST intrinsic connectivity with: subcortical regions (amygdala, nucleus accumbens, anterior hippocampus, hypothalamus) and cortical regions (anterior insula, ventromedial prefrontal cortex, anterior cingulate cortex), as well as the posterior cingulate cortex, visual cortex, and parietal cortex (Avery et al., 2014, Torrisi et al., 2015, Gorka et al., 2018, Tillman et al., 2018, Berry et al., 2021). This network has been associated with emotion, stress, and reward processing (for examples see Crestani et al., 2013, Dabrowska et al., 2016, Kinnison et al., 2012), suggesting that the BNST network is of critical importance to study.

While studies in adults have been critical for identifying the architecture of the BNST intrinsic network, little is known about the development of this network. Several lines of research suggest that the BNST and its network may change across adolescence. Significant changes in emotional processing, reward processing, and social interactions—which are modulated by the BNST—occur during adolescence. For example, adolescents often experience relatively rapid emotional state transitions, more complex and stronger emotional responses to triggers, and decreased ability to regulate emotions (for review see McLaughlin et al., 2015). Adolescence is also a period of social change, where adolescents spend less time with their family members and more time with peers (Larson and How, 2001). Rapid changes in emotions and social structure during adolescence are thought to contribute to vulnerability to psychopathology that often emerges during this period (Angold et al., 1998, Kessler et al., 2005, Solmi et al., 2022), suggesting that brain changes in key regions – such as the BNST – occurring during this period could underlie this risk. Furthermore, a neuroimaging study of unpredictable threat anticipation in children aged 8–10 years suggests a developmental shift in BNST function: children show different brain responses to unpredictable threat than adults (Feola et al., 2021). Specifically, studies of unpredictable threat anticipation report robust BNST activation in adults (Clauss et al., 2019, Brinkmann et al., 2018, Klumpers et al., 2017, Hur et al., 2020) and adolescents (Hur et al., 2025). However, children failed to show BNST activation to unpredictable threat and, instead, showed heightened amygdala activation (Feola et al., 2021). Thus, multiple lines of converging evidence suggest that the BNST may undergo developmental shifts during middle childhood to early adulthood.

To date, there have been limited investigations of BNST intrinsic connectivity in youth. Niu and colleagues (Niu et al., 2025) recently examined BNST intrinsic functional connectivity in one month old infants. A whole brain analysis revealed clusters of significant BNST connectivity with the amygdala, hippocampus, and ventral striatum, suggesting that key BNST connections are established very early in life. Feola and colleagues (Feola et al., 2021) performed a BNST intrinsic connectivity analysis in individuals aged 8–10 years, and showed significant intrinsic connectivity with the amygdala, hippocampus, caudate, insula, thalamus, ventromedial prefrontal cortex (vmPFC), rostral anterior cingulate cortex (rACC) and dorsal anterior cingulate cortex (dACC). While this BNST intrinsic network was visually similar to the adult network, a formal comparison by age was not performed. These findings suggest that the BNST network may undergo a significant expansion to cortical regions between infancy and middle childhood. Jin and colleagues (Jin et al., 2023) investigated age-related changes in BNST intrinsic connectivity in 9–18 year olds (healthy and socially anxious) during movie-watching. A follow-up analysis of connectivity during a rest condition found no significant age or group effects on BNST connectivity during rest. However, the analysis of resting state data was limited to six brain regions that showed BNST connectivity during movie watching. Thus, there remains a significant gap in understanding the development of BNST intrinsic connectivity.

The BNST has been described as a sexually dysmorphic region, with early post-mortem human and rodent studies demonstrating larger BNST volume in men (Allen and Gorski, 1990) and male rats (Hines et al., 1992). However, a recent study estimating volume from MRI scans in humans showed that the sex differences are quite small (Berry et al., 2024). For BNST structural connectivity, adult women have stronger connectivity with multiple other brain regions compared with men (Avery et al., 2014, Flook et al., 2020). For BNST resting state fMRI studies, the results are mixed. A study of BNST-amygdala effective connectivity at rest showed stronger connectivity in men, compared with women, from two amygdala subnuclei to the BNST (Hofmann and Straube, 2019). However, a whole-brain BNST resting state fMRI study found sex differences were limited; men exhibited stronger BNST-putamen connectivity and women exhibited stronger BNST-thalamus connectivity (Avery et al., 2014). There is minimal information about BNST sex differences during childhood and adolescence, but there are preliminary findings in a similar region, the amygdala. A resting state study of amygdala subnuclei connectivity in children and adolescents found that boys and girls had opposite patterns of connectivity with increasing age across multiple regions; for example, connectivity between the superficial amygdala subnucleus and dorsomedial prefrontal cortex increased with age for girls and decreased with age for boys (Alarcón et al., 2015). Thus, while there are mixed findings regarding sex differences in BNST structure and function in adulthood, sex differences may exist during childhood and adolescence.

The purpose of the present study was to characterize the development of the BNST intrinsic functional network using a large, heterogeneous sample of children and young adults (8–23 years). This study uses a cross-sectional neuroimaging cohort from a publicly available dataset, the Philadelphia Neurodevelopmental Cohort (PNC; Satterthwaite, 2014, Satterthwaite et al., 2016). We hypothesize that BNST intrinsic connectivity will change across development. Based on developmental findings in a similar region, the amygdala (Qin et al., 2012, Gabard-Durnam et al., 2014), we propose that children, compared with young adults, will show stronger connectivity between the BNST and subcortical regions and weaker connectivity with prefrontal cortical (PFC) regions. The PFC has both structural and functional connections with the BNST in adults (Avery et al., 2014, Gorka et al., 2018, Tillman et al., 2018, Torrisi et al., 2019) and is one of the last regions to develop (for review see Kolk and Rakic, 2022). We also tested for sex differences, based on evidence that the BNST may be sexually dimorphic (Hines et al., 1992, Allen and Gorski, 1990).

## 2 Methods

### 2.1 Participants

Data used in this study were collected as part of the Philadelphia Neurodevelopmental Cohort (PNC; Satterthwaite, 2014, Satterthwaite et al., 2016), for which participants were recruited from a pediatric health care network in Pennsylvania, New Jersey, and Delaware. The PNC sample includes a community sample of non-treatment seeking youth. The study was designed to capture the full range of individual variability present in youth, consistent with epidemiological studies. Its inclusive recruitment strategy, without restriction to “typically developing” participants, provides a more representative view of developmental processes, making it well suited for examining BNST development. Because the goal of this study was to determine BNST connectivity reflective of a diverse population, the inherent heterogeneity of the original data was maintained. Original study procedures were approved by the Institutional Review Boards of the University of Pennsylvania and the Children’s Hospital of Philadelphia. Data for the Philadelphia Neurodevelopmental Cohort (PNC) were obtained through the Database of Genotypes and Phenotypes platform (phs000607.v3.p2; project #35550; JUB).

Participants included individuals who had resting state fMRI data acquired as part of the PNC data collection (Satterthwaite et al., 2014).The final sample included *n* = 1134 participants (46 % boys, determined by self-report) ages 8 – 23 years (M = 15.77, SD = 3.36, see Supplemental Figure 1). In the sample, the distribution of self-reported race was 47 % European American, 45 % African American, 7 % multiple reported, and 0.44 % unknown or did not provide answer; ethnicity was reported as 5 % Hispanic. Parental education (e.g., years of schooling) was reported as: maternal education (M = 14.3 yrs, SD = 2.47, data missing for *n* = 16) and paternal education (M = 14.05 yrs, SD = 2.71, data missing for *n* = 90).

### 2.2 Imaging data collection

Imaging data were acquired using a Siemens 3 T scanner with a 32-channel head coil. The resting state scans were 6.3 min with the following parameters: TR/TE 3000/32 ms, flip = 90°, FOV = 192 × 192, slice thickness 3 mm, and voxel size 3 × 3 × 3 mm. For more details see (Satterthwaite et al., 2014).

### 2.3 Imaging preprocessing

Preprocessing steps included the following: motion realignment; rigid body coregistration to the T1-weighted structural image; bandpass filter 0.01–0.10 Hz; and confound removal *via* regression (six estimated motion parameters, and six principal components from the white matter and CSF compartment). The T1 structural image was registered nonlinearly to the IXI155 MNI152 space template using the CAT12 toolbox (version 12.5). Functional images were resampled to 2 mm isotropic voxels. The images were not smoothed to provide greater spatial specificity and avoid issues with partial volume effects in the small regions of interest. The effective voxel size in the final images was 2.2 mm (Dong et al., 2000). For details see https://github.com/baxpr/connprep/tree/v2.1.0. Resting state scans were excluded if mean framewise displacement (FD) exceeded 0.5 mm. The final number of participants with resting-state scans that met criteria was *n* = 1134. Average mean FD for the final sample was 0.18 mm with a standard deviation of 0.10 mm; the mean 95th percentile for voxel displacement was 0.25 mm with a standard deviation of 0.20 mm.

### 2.4 Imaging analysis

Resting state fMRI data were analyzed using SPM (v12; Ashburner et al., 2020). The seeds were the left and right BNST, anatomically defined using a previously published 3 T mask (Avery et al., 2014, Theiss et al., 2017), which has been used successfully in children (Feola et al., 2021). Whole brain functional connectivity maps were created using extracted mean time series from the left and right BNST separately (https://github.com/baxpr/mniconn/tree/v3.1.2). The Fisher transformed correlation-coefficients were used as the outcome variables.

### 2.5 Regions of interest

We first performed a region of interest (ROI) analysis of a putative BNST network based on studies of BNST structural and functional connectivity in adults (Avery et al., 2014, Flook et al., 2020). For the region of interest analysis, average BNST connectivity values were extracted for regions of the BNST network: amygdala, anterior hippocampus, anterior insula, hypothalamus, nucleus accumbens, and vmPFC. The hypothalamus was created using the same methods as the BNST. The Harvard-Oxford Atlas (Frazier et al., 2005, Desikan et al., 2006) templates (50 % probability) were used for the nucleus accumbens, amygdala, and hippocampus. The hippocampus was then manually segmented into the anterior hippocampus using the uncus as the anterior/posterior boundary. The anterior insula was defined using a mask developed by Farb (Farb et al., 2013) which was previously validated as having structural connectivity with the BNST (Flook et al., 2020). Finally, the ventromedial prefrontal cortex (vmPFC) mask was defined based on a 10 mm sphere surrounding the peak point of vmPFC-BNST connectivity from our previous study [x = 0, y = 43, z = -9] (Avery et al., 2014).

### 2.6 Statistical analyses

Regression analyses for the region of interest approach were performed in R using the *lmer* package (Bates et al., 2015), with age, sex, and age x sex as factors. To investigate non-linear effects, which may capture changes during a specific time period (e.g., adolescence), we included linear, quadratic, and cubic effects. The *poly* function in R was used to create orthogonal polynomial terms, which have the benefit of reducing multicollinearity, ensuring the unique effect of each term, and producing more stable model estimates. The model included linear, quadratic, and cubic effects of age and interactions with sex. Only the linear effects were significant, therefore the final models were run with only linear effects.

Because ROI approaches may provide a limited perspective, we also included a whole-brain exploratory analysis, using the BNST as a seed, to assess brain-wide changes in BNST connectivity. For the whole brain analysis, voxel-wise general linear models (GLMs) were implemented in SPM with age, sex, and age x sex as predictor variables and scanner motion (mean FD) as a covariate. The model included linear, quadratic and cubic effects of age and interactions with sex. Only the linear effects were significant, therefore the final models were run with only linear effects. Age was mean-centered prior to model estimation. Sex was specified using effect coding (-1, 1). This approach allowed us to evaluate both age-independent and age-dependent effects. Age-independent effects were measured using model intercept, which represents the variability in the absence of age-related modulation. Age-related effects were identified by testing linear associations with age, accounting for sex and motion. A voxel p-value of < .001 and cluster size (k) of 26 provided a FWE cluster correction of α = 0.05 (using SPM cluster threshold). For significant interactions, we computed Pearson’s correlation to characterize the strength and direction of the effect.

## 3 Results

### 3.1 BNST network region of interest analyses

**Fig. 1:**
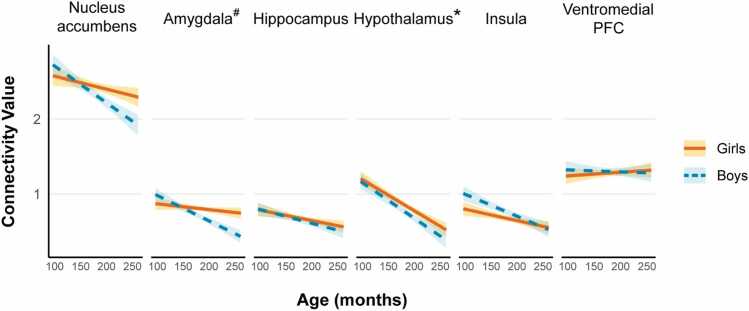
**Age related changes in BNST network intrinsic connectivity by sex.** This figure illustrates the linear age-related patterns of BNST connectivity with regions of the BNST network: nucleus accumbens, amygdala, hippocampus, hypothalamus, insula, and ventromedial prefrontal cortex (PFC). Confidence intervals (95 %) are shown by shaded areas. # indicates an age x sex interaction; * indicates a main effect of age.

BNST connectivity with the core network regions by age and sex is shown in Fig. 1 and in Supplemental Table 1. There were significant changes with age for two regions. BNST-hypothalamus connectivity decreased linearly with age (*t*(1126) = -4.59, *d* = 0.12, *p* < 0.001). BNST-amygdala connectivity had a main effect of sex (*t(*1126) = -2.55, *p* = 0.02), which was qualified by a linear age x sex interaction (*t*(1126) = -2.43, *d* = 0.15, *p* = 0.02). BNST-amygdala connectivity showed a significant linear decrease for boys (*r* = -0.13, *p* < 0.001), with no significant change in girls (*r* = -0.03, *p* = 0.28).

### 3.2 Whole-brain connectivity

**Fig. 2:**
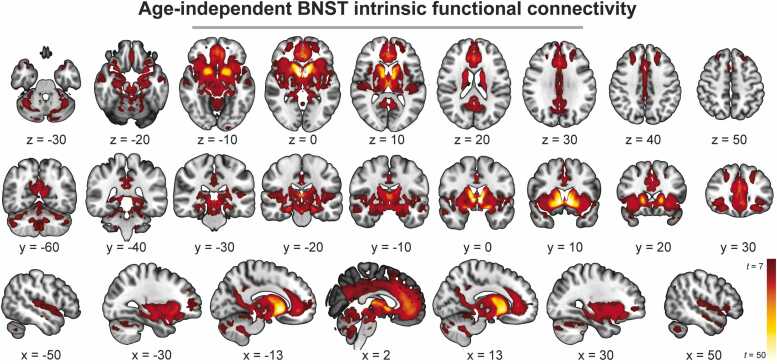
**Age-independent BNST intrinsic connectivity.** Image shows connectivity pattern from the left BNST seed, which was similar to the right BNST seed. For visualization purposes, values were thresholded at t = 7. *BNST = bed nucleus of the stria terminalis*.

*Age- and sex-independent connectivity.* The BNST showed strong intrinsic functional connectivity across a distributed network of brain regions, independent of age and sex (Fig. 2). The significant cluster was very large and encompassed many brain regions (clusters of k = 106,936 for the left BNST and k = 111,331 for the right BNST). To provide more specific anatomical information, we identified the top 20 local maxima using a 3 mm 3D kernel. Each local maximum was then assigned to a region based on the AAL atlas (Rolls et al., 2020), with the most significant vertex from each region reported (Table 1). Across the sample, the BNST had diffuse connectivity with the hippocampus, caudate, putamen, thalamus, orbitofrontal cortex (OFC), and anterior cingulate cortex (ACC).

**Table 1:** Local maxima within age-independent cluster of BNST intrinsic connectivity.

	Local Maxima (x, y, z)	Peak (Z)
Left BNST		
BNST	-6, 2, 0	11.25
Caudate Putamen Thalamus Anterior Cingulate Cortex Medial Orbitofrontal Cortex Hippocampus	8, 4, 0 18, 8, −8 2, −18, 8 0, 38,6 -10, 44, −6 -20, −18, −16	10.42 9.97 9.65 9.54 9.49 9.29
Right BNST		
BNST Caudate Putamen Thalamus Anterior Cingulate Cortex Medial Orbitofrontal Cortex	6, 4, 0 14, 14, −2 18, 8, −8 2, −14, 8 0, 42, 2 0, 48, −6	11.26 10.14 9.95 9.62 9.60 9.54

Note: Cluster sizes are left BNST k = 106,936 and right BNST k = 111,331

**Fig. 3:**
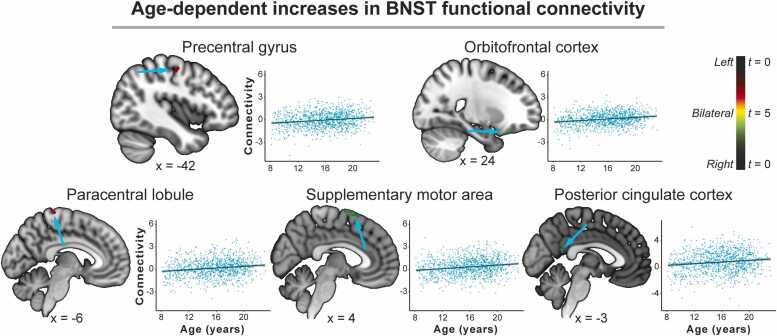
**Age-dependent increases in BNST connectivity.** Left and right BNST connectivity with the precentral gyrus, orbitofrontal cortex, paracentral lobule, supplementary motor area, and posterior cingulate cortex increased with age. Color bar: Left BNST connectivity is represented by green, right BNST connectivity by red, and bilateral BNST by yellow. BNST = bed nucleus of the stria terminalis.

*Age-dependent increases in connectivity.* Age-dependent increases in left and right BNST intrinsic functional connectivity were discovered in the precentral gyrus, posterior cingulate cortex, and posterior OFC. Specifically, there was a positive effect of age on connectivity between the left BNST and the precentral gyrus, such that connectivity was stronger in the older participants. In the right BNST, there was a significant positive effect of age on connectivity with regions of the supplementary motor area, posterior cingulate cortex, and posterior OFC, such that connectivity was stronger in older participants. For full results, see Table 2 and Fig. 3.

**Table 2:** Cluster characteristics of age-dependent BNST intrinsic connectivity.

	Cluster size	Peak voxel (x, y, z)	Peak (Z)	Correlation (*r*)
Age-dependent increases				
Left BNST				
Precentral gyrus	52	-44, −12, 54	4.51	0.17
Paracentral lobule	51	-10, −30, 66	4.22	0.16
Right BNST				
Posterior orbitofrontal cortex	62	30, 28, −26	4.40	0.21
Supplementary motor area	39	2, 16, 64	4.21	0.17
Posterior cingulate cortex	27	0, −52, 16	4.21	0.13
Age-dependent decreases				
Left BNST				
Nucleus accumbens (nucleus accumbens, lentiform nucleus, pallidum, and ventral BNST)	278	6, 4, −8	6.81	-0.26
Medial orbitofrontal cortex	27	-20, 18, −14	5.91	-0.20
Caudate	114	-16, 20, 12	4.98	-0.24
Posterior hippocampus	51	32, −38, −4	4.76	-0.18
	27	-24, −42, 0	4.54	-0.22
Anterior cingulate cortex	46	12, 36, 8	4.20	-0.23
Right BNST				
Nucleus accumbens (nucleus accumbens, lentiform nucleus, pallidum, and ventral BNST)	356	6, 4, −8	∞	-0.03
Anterior cingulate cortex, caudate	291	14, 32, 16	5.63	-0.27
Mid-cingulate cortex	61	6, −20, 30	5.49	-0.17
Precuneus	95	12, −64, 44	4.96	-0.15
Thalamus and caudate	84	0, 2, 14	4.56	-0.24
Thalamus	65	12, −22, 18	4.49	-0.23
Midbrain, substantia nigra	31	-6, −12, −14	4.49	-0.21
Anterior hippocampus	34	-26, −8, −16	4.39	-0.17
*Effect sizes (r) are provided to characterize the direction and strength of the linear effects of age.*

**Fig. 4:**
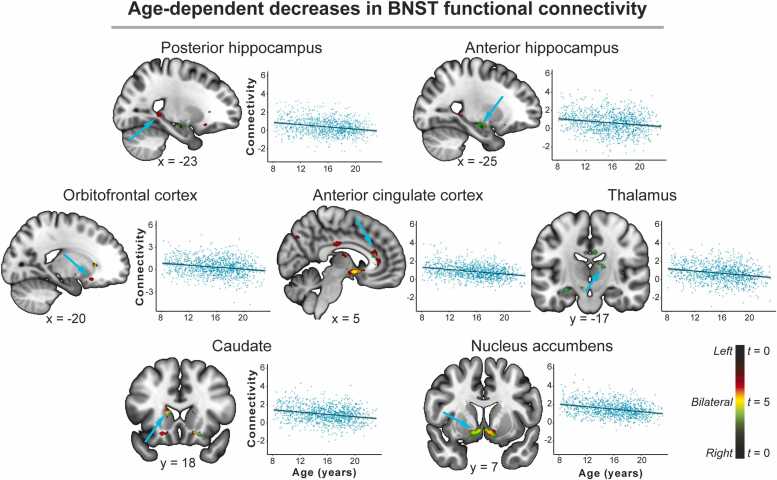
**Age-dependent decreases in BNST connectivity.** Left and right BNST connectivity with the posterior hippocampus, anterior hippocampus, thalamus, orbitofrontal cortex, anterior cingulate cortex, and nucleus accumbens decreased with age across the sample. Color bar: Left BNST connectivity is represented by green, right BNST connectivity by red, and bilateral BNST by yellow. BNST = bed nucleus of the stria terminalis.

*Age-dependent decreases in connectivity.* Age-dependent decreases for the left and right BNST were also revealed, predominantly in subcortical brain regions. For both the left and right BNST, there was a negative effect of age such that connectivity was stronger in the younger participants with a large subcortical cluster (referred to as the nucleus accumbens, which contained the nucleus accumbens, lentiform nucleus, pallidum, and ventral BNST), and the ACC. The left BNST also showed a negative effect of age with the posterior hippocampus and medial OFC. For the right BNST, a negative effect of age was also found in the anterior hippocampus, thalamus, caudate, brain stem, midcingulate cortex, and precuneus. For full results, see Table 2 and Fig. 4.

*Sex-dependent BNST connectivity.* We also tested for sex effects (sex, age x sex interaction) in left and right BNST connectivity. For right BNST connectivity, there were significant main effects of sex in the parietal cortex (x = -38, y = -54, z = 36; k = 31, Z = 4.19) and the occipital cortex (x = 4, y = -90, z = 4; k = 33, Z = 4.17). For the occipital cortex, the main effect of sex was qualified by a significant age x sex interaction; boys displayed stronger connectivity with increasing age (*r* = 0.19), while girls displayed weaker connectivity with increasing age (*r* = -0.10). There were no significant sex effects for left BNST connectivity.

## 4 Discussion

The main goal of this study was to investigate age-related changes in BNST intrinsic functional connectivity between middle childhood and early adulthood in a community-based sample. Because the PNC was recruited from the community rather than clinical or convenience samples, the sample reflects the natural heterogeneity of youth development. The sample’s diversity allows for the characterization of BNST maturation within a context that more closely approximates the population. There were three main findings. First, we identified robust age-independent BNST connectivity in a distributed network including the hippocampus, caudate, putamen, thalamus, anterior cingulate cortex (ACC), and orbitofrontal cortex (OFC). Second, we discovered age-dependent changes in intrinsic connectivity, largely characterized by age-related increases in BNST connectivity with motor and control regions and age-related decreases in BNST-subcortical connectivity. Third, we found evidence for sex-dependent effects in age-related changes in BNST connectivity with amygdala and occipital cortex.

Across the sample, there was an age-independent pattern of BNST connectivity across the brain, which included connectivity with the hippocampus, caudate, putamen, thalamus, ACC, and OFC. The pattern of whole-brain BNST intrinsic connectivity across the sample was largely consistent with previously described BNST connectivity in adults (Avery et al., 2014, Torrisi et al., 2015, Gorka et al., 2018, Tillman et al., 2018) and a distinct but smaller sample of children aged 8–12 years that also identified BNST intrinsic connectivity with the thalamus and ACC (Feola et al., 2021). An age-independent BNST network suggests that BNST intrinsic connectivity is largely established by middle childhood.

The BNST connectivity pattern described is similar to previous connectivity studies of the amygdala, another part of the extended amygdala, that is more widely studied. For example, a cross-sectional study (participants aged 4–23 years) of amygdala intrinsic connectivity demonstrated a broad and stable intrinsic network, with age-related increases in connectivity with the medial PFC and age-related decreases with the insula, posterior cingulate cortex, and parahippocampal gyrus (Gabard-Durnam et al., 2014). Furthermore, a follow-up study in three-month to five-year-olds found that, generally, whole-brain amygdala connectivity was already intact, suggesting that these networks develop early in infancy and are fine-tuned with development (Gabard-Durnam et al., 2018). Taken together, these findings suggest a similar developmental trajectory between the BNST and amygdala, based on substantial overlap between prior studies of BNST connectivity in adults and our findings of whole-brain BNST connectivity in a younger sample.

We also reported on age-dependent changes in BNST intrinsic connectivity; connectivity with motor and planning regions strengthened with age, while connectivity with subcortical regions weakened with age. We discovered age-related increases in BNST connectivity with distant motor and control regions, including the precentral gyrus (primary motor cortex), superior frontal gyrus (supplementary motor area), posterior cingulate cortex, and posterior OFC. Stronger BNST connectivity with motor and planning regions is consistent with findings from previous studies in adults (Avery et al., 2014, Tillman et al., 2018), and are intriguing when considering animal studies examining BNST connectivity with the OFC and the motor cortex. For example, one study in primates (Fox et al., 2010) demonstrated that OFC lesions impaired BNST metabolism, measured by decreases in FDG PET. Findings in rodents also reveal that connections from the motor cortex to BNST-projecting insula neurons control behavioral responses to restraint stress (Luchsinger et al., 2021). These findings in animals suggest connections from these motor and control regions can alter BNST activity and impact stress responses, which could have implications in humans for stress-related psychopathology.

We also discovered age-related decreases in BNST connectivity. In the BNST network analysis, we identified a negative age effect such that younger individuals had significantly stronger connectivity with the hypothalamus than older individuals. The whole brain analysis also showed that younger individuals had stronger BNST connectivity with subcortical regions, including the nucleus accumbens, midbrain, lentiform nucleus, thalamus, amygdala, and ACC, replicating prior studies showing these subcortical connections in human adults and animal models (for examples see Avery et al., 2014, Dong and Swanson, 2006, Dong et al., 2000, Tillman et al., 2018, Torrisi et al., 2015). These findings suggest that this subcortical-BNST connectivity emerges early and, while weakening somewhat over the course of the transition from childhood to adulthood, remains a core network into adulthood. Interestingly, these findings demonstrated an age-associated shift in BNST intrinsic connectivity from more proximal, subcortical regions, to more distal, regulatory cortical regions. BNST connectivity shifting from local to distant is an important finding, building on prior studies showing connectivity shifts from short to long-range with age (e.g Di Martino et al., 2014, Dosenbach et al., 2010, Ernst et al., 2015, Fair et al., 2009, Fair et al., 2007, Langen et al., 2018, Rubia, 2013). Together, these data suggest that BNST-subcortical connectivity is not lost with age; rather, it is fine-tuned over the course of development.

In addition to our *a priori* hypotheses related to age-dependent connectivity differences, we also explored sex differences. Our exploratory investigation of sex differences revealed very few differences. Seminal studies of BNST volume in rats (Hines et al., 1992) and post-mortem studies in humans (Allen and Gorski, 1990) suggest sexual dimorphism, with larger BNST volume in males compared to females (although see Berry et al., 2024). Our finding of limited sex differences is consistent with this recent MRI study and prior studies of BNST intrinsic connectivity in human adults that report few sex differences (Avery et al., 2014, Torrisi et al., 2015, Tillman et al., 2018). In the present study, there were two patterns observed only in boys: weakened BNST-amygdala connectivity and strengthened BNST- occipital cortex connectivity. The significance of sex differences remains unclear, as findings are often limited or inconsistent. Few studies have directly examined the effect of sex in development (for recent review see Uddin et al., 2025), fewer still in BNST development. This gap in the literature underscores the need for further longitudinal research to elucidate how sex-linked biological and environmental factors may influence BNST intrinsic connectivity across development. Moreover, the developmental timing of hormonal changes, including those associated with adrenarche and puberty, may contribute to subtle sex-specific patterns in BNST connectivity that are not easily detectable in cross-sectional samples. As such, while our findings provide preliminary evidence for modest sex-related effects, particularly in young boys, they should be interpreted with caution. Future studies with larger samples, longitudinal designs, and direct measurement of pubertal status will be critical to disentangling the complex interplay between sex, development, and BNST connectivity.

Furthermore, there are additional noted limitations and important future directions of the present study. First, the data used in this study have limitations inherent to cross-sectional analysis, including cohort effects and inability to establish causal relations. Longitudinal investigations will be crucial for characterizing BNST network connectivity changes and potential factors that could impact developmental trajectories of BNST network connectivity (e.g., sex). Second, the study sample does not include early childhood, which could represent an important time period for BNST development. Future studies of infancy through middle childhood will be critical to characterizing early development of BNST connectivity. Third, the PNC sample was designed to be inclusive of a wide range of individuals from a community-based sample. The heterogeneity of the sample provides important generalizability but could also obscure subgroups where development of BNST connectivity is altered. Although the sample size is relatively large, reliable measures within subgroups are likely to be underpowered, given the myriad possible permutations of symptoms, diagnoses, and phenotypes. The BNST is implicated in several psychopathologies, including anxiety and substance use disorders that often begin to emerge during adolescence. A limitation of the current study is that we were unable to investigate subgroups meeting criteria for psychopathology. Future research should focus on targeted cohorts to explore these subgroups, allowing for a deeper understanding of how specific factors influence BNST connectivity and its developmental trajectories.

In summary, this study demonstrates that BNST intrinsic connectivity is largely established prior to adolescence and that an important shift from connectivity with local subcortical regions to more distant motor and control regions occurs across development. These findings lay the foundation of BNST network connectivity across a critical developmental period, therefore allowing us to begin inquiries into whether BNST network development differs in youth with psychiatric disorders.

## Funding

This work was supported by the 10.13039/100000002National Institutes of Health (NIAAA F30 AA027418 to EF, NIAAA F32 AA032170 to NLZ)

## CRediT authorship contribution statement

**Flook Elizabeth:** Writing – review & editing, Writing – original draft, Visualization, Methodology, Formal analysis, Conceptualization. **Jennifer Urbano Blackford:** Writing – review & editing, Visualization, Supervision, Methodology, Funding acquisition, Formal analysis, Data curation, Conceptualization. **Nicole L. Zabik:** Writing – review & editing, Visualization, Formal analysis. **Brandee Feola:** Writing – review & editing, Conceptualization. **Baxter Rogers:** Writing – review & editing, Resources, Methodology, Data curation.

## Data Access

Data were obtained from dbGap (phs000607.v3.p2; project #35550). Support for the collection of the data for Philadelphia Neurodevelopment Cohort (PNC) was provided by grant RC2MH089983 awarded to Raquel Gur and RC2MH089924 awarded to Hakon Hakonarson. Subjects were recruited and genotyped through the Center for Applied Genomics (CAG) at The Children's Hospital in Philadelphia (CHOP). Phenotypic data collection occurred at the CAG/CHOP and at the Brain Behavior Laboratory, University of Pennsylvania.

## Declaration of Competing Interest

The authors declare that they have no known competing financial interests or personal relationships that could have appeared to influence the work reported in this paper.

## Data Availability

The authors do not have permission to share data.
