# Supplementary material for: The development of BNST intrinsic functional connectivity from 8 to 23 years of age: A PNC cohort study
Source: Dev Cogn Neurosci. 2025 Dec 19;78:101661. doi: 10.1016/j.dcn.2025.101661 (PMC12856425; doi:10.1016/j.dcn.2025.101661)
Supplement: Supplementary file 1 — Supplementary material [file mmc1.docx]

**Supplemental Table 1. Effects of age and sex on intrinsic connectivity in BNST network**

|  | **Beta weight (SE)** | **t value** | **df** | **Cohen’s d** | **p value** |
| --- | --- | --- | --- | --- | --- |
| **Accumbens** |  |  |  |  |  |
| Age | -2.09 (1.52) | -1.37 | 1126 | 0.08 | 0.17 |
| Sex | -0.12 (0.07) | -1.77 | 1126 | 0.11 | 0.08 |
| Age x sex | -4.02 (2.25) | -1.79 | 1126 | 0.11 | 0.07 |
|  |  |  |  |  |  |
| **Amygdala** |  |  |  |  |  |
| Age | -0.94 (0.90) | -1.04 | 1126 | 0.06 | 0.30 |
| **Sex** | **-0.10 (0.04)** | **-2.55** | **1126** | **0.15** | **0.01** |
| **Age x sex** | **-3.25 (1.34)** | **-2.43** | **1126** | **0.15** | **0.02** |
|  |  |  |  |  |  |
| **Hippocampus** |  |  |  |  |  |
| Age | -1.68 (0.97) | -1.74 | 1126 | 0.10 | 0.08 |
| Sex | -0.03 (0.04) | -0.69 | 1126 | 0.04 | 0.49 |
| Age x sex | -0.53 (1.43) | -0.37 | 1126 | 0.02 | 0.71 |
|  |  |  |  |  |  |
| **Hypothalamus** |  |  |  |  |  |
| **Age** | **-5.15 (1.12)** | **-4.59** | **1126** | **0.12** | **<0.001** |
| Sex | -0.09 (0.05) | -1.78 | 1126 | 0.01 | 0.07 |
| Age x sex | -0.74 (1.66) | -0.44 | 1126 | 0.11 | 0.66 |
|  |  |  |  |  |  |
| **Insula** |  |  |  |  |  |
| Age | -1.96 (1.11) | -1.76 | 1126 | 0.12 | 0.08 |
| Sex | 0.08 (0.05) | 1.69 | 1126 | 0.04 | 0.09 |
| Age x sex | -1.70 (1.65) | -1.03 | 1126 | 0.03 | 0.30 |
|  |  |  |  |  |  |
| **vmPFC** |  |  |  |  |  |
| Age | 0.60 (1.31) | 0.46 | 1126 | 0.13 | 0.65 |
| Sex | 0.02 (0.06) | 0.30 | 1126 | 0.05 | 0.76 |
| Age x sex | -0.83 (1.95) | -0.42 | 1126 | 0.17 | 0.67 |
