# Supplementary figures and images for: The development of BNST intrinsic functional connectivity from 8 to 23 years of age: A PNC cohort study
Source: Dev Cogn Neurosci. 2025 Dec 19;78:101661. doi: 10.1016/j.dcn.2025.101661 (PMC12856425; doi:10.1016/j.dcn.2025.101661)

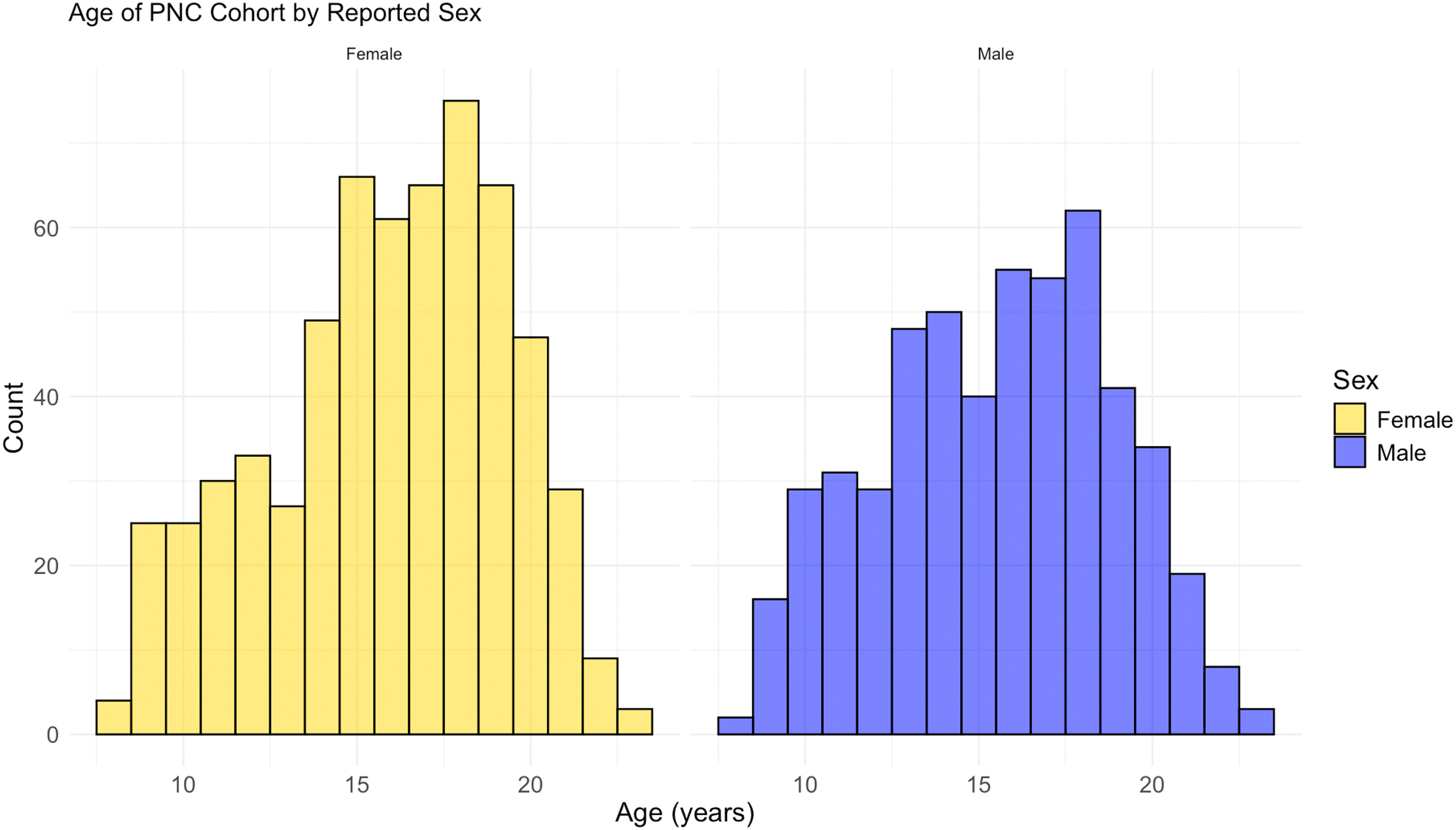

Supplement: Supplementary file 2 — Supplementary material [file mmc2.jpg]
